# Optimizing cryopreservation conditions for use of fucosylated human mesenchymal stromal cells in anti-inflammatory/immunomodulatory therapeutics

**DOI:** 10.3389/fimmu.2024.1385691

**Published:** 2024-03-28

**Authors:** Jesús I. Gil-Chinchilla, Carlos Bueno, Carlos M. Martínez, Ana Ferrández-Múrtula, Ana M. García-Hernández, Miguel Blanquer, Mar Molina-Molina, Agustín G. Zapata, Robert Sackstein, Jose M. Moraleda, David García-Bernal

**Affiliations:** ^1^ Hematopoietic Transplant and Cellular Therapy Unit, Instituto Murciano de Investigación Biosanitaria (IMIB) Pascual Parrilla, University of Murcia and Virgen de la Arrixaca University Hospital, Murcia, Spain; ^2^ Experimental Pathology Unit, Instituto Murciano de Investigación Biosanitaria (IMIB) Pascual Parrilla, University of Murcia, Murcia, Spain; ^3^ Department of Medicine, University of Murcia, Murcia, Spain; ^4^ Department of Cell Biology, Complutense University, Madrid, Spain; ^5^ Department of Translational Medicine, and the Translational Glycobiology Institute, Herbert Wertheim College of Medicine, Florida International University, Miami, FL, United States; ^6^ Department of Biochemistry, Molecular Biology, and Immunology, University of Murcia, Murcia, Spain

**Keywords:** mesenchymal stromal cells, exofucosylation, HCELL, E-selectin ligand, cryopreservation, immunomodulation, cell therapy

## Abstract

Mesenchymal stem/stromal cells (MSCs) are being increasingly used in cell-based therapies due to their broad anti-inflammatory and immunomodulatory properties. Intravascularly-administered MSCs do not efficiently migrate to sites of inflammation/immunopathology, but this shortfall has been overcome by cell surface enzymatic fucosylation to engender expression of the potent E-selectin ligand HCELL. In applications of cell-based therapies, cryopreservation enables stability in both storage and transport of the produced cells from the manufacturing facility to the point of care. However, it has been reported that cryopreservation and thawing dampens their immunomodulatory/anti-inflammatory activity even after a reactivation/reconditioning step. To address this issue, we employed a variety of methods to cryopreserve and thaw fucosylated human MSCs derived from either bone marrow or adipose tissue sources. We then evaluated their immunosuppressive properties, cell viability, morphology, proliferation kinetics, immunophenotype, senescence, and osteogenic and adipogenic differentiation. Our studies provide new insights into the immunobiology of cryopreserved and thawed MSCs and offer a readily applicable approach to optimize the use of fucosylated human allogeneic MSCs as immunomodulatory/anti-inflammatory therapeutics.

## Introduction

Mesenchymal stem/stromal cells (MSCs) comprise a heterogeneous cell population that resides in a wide variety of tissues including bone marrow, adipose tissue, umbilical cord, amniotic membrane and dental pulp (1). Despite this recognized heterogeneity, MSCs are more homogeneous than other stem cell types such as induced pluripotent stem cells (iPSCs), hematopoietic stem cells or neural stem cells. MSCs possess the ability to self-renew and differentiate into mesodermal tissues (e.g., bone, adipose tissue and cartilage), regulate tissue homeostasis through the production of trophic factors, and have anti-inflammatory and immunomodulatory properties (e.g., suppress the proliferative response of activated immunocompetent cells in a dose-dependent manner) (2–5). Due to these multiple biological effects, MSCs are becoming a widely used cellular therapy, with ongoing clinical trials investigating their therapeutic effects in a variety of acute and chronic inflammatory disorders and degenerative diseases, including graft-versus-host disease, COVID-19, osteoporosis, Crohn’s disease, and cardiac decompensation (6–10).

Following intravenous administration, MSC migration to inflamed/damaged tissue(s) is very limited, and only a small proportion of systemically administered MSCs reach their intended target(s) due to mechanical entrapment in small diameter pulmonary and hepatic blood vessels, as a result of their large cell size, especially after their *ex vivo* culture, and their important deficits in the expression of homing molecules (11–16). Indeed, MSCs prominently express CD44, but not its sialofucosylated glycovariant known as HCELL (Hematopoietic Cell E-/L-selectin Ligand). By definition, HCELL is CD44 that displays the sialofucosylated tetrasaccharide determinant known as sialyl Lewis X (sLe^X^ or CD15s) and this glycovariant is a potent mediator of the critical first steps in the migration of blood-borne cells to E-selectin expressing endothelial cells within the microvasculature of the skin, bone marrow and inflamed tissues (17–19). Natively, MSC CD44 is decorated with sialylated lactosamines, lacking only the presence of fucose in α1,3)-linkage to the lactosaminyl moiety (specifically, to the N-acetylglucosamine) required for the construction of the sLe^X^ determinant (20–22). Sackstein et al. developed a method to transiently convert the CD44 molecule into its HCELL glycovariant by selective enzymatic α(1,3)-fucosylation of the MSC surface (“exofucosylation”), thereby programming MSC homing to E-selectin-bearing endothelial beds (21, 23). Exofucosylated MSCs (FucMSCs) can be manufactured under GMP conditions and have demonstrated an improved *in vivo* migratory capacity to target tissues after systemic administration in different preclinical models, as well as greater anti-inflammatory potency both *in vivo* and *in vitro* (5, 20–22, 24).

Human MSC manufacturing for clinical applications frequently demands cryopreservation and transport of the cells from the GMP manufacturing facility to the site of thawing and infusion into the patient. In particular, the applicability of cryopreserved FucMSCs for anti-inflammatory/immunomodulatory therapeutics remains unknown (25–27), yet it is well recognized that cryogenic procedures can irreversibly damage cells, severely compromising their viability and functionality after thawing (28). In the present work, we investigated how different cryopreservation and thawing conditions impact the anti-inflammatory/immunomodulatory properties of human bone marrow and adipose tissue-derived FucMSCs, in order to design optimized protocols for clinical application of these cells in acute/chronic inflammatory and degenerative pathologies.

## Materials and methods

### Isolation and culture of human bone marrow and adipose tissue-derived mesenchymal stromal cells

Human bone marrow and adipose tissue samples (N=5 per tissue) were obtained from healthy donors after written informed consent under protocols approved by the Institutional Review Boards of the Virgen de la Arrixaca University Hospital (Murcia, Spain). Bone marrow was collected by iliac crest aspiration in syringes containing 20U/mL sodium heparin (Laboratorios Rovi, Madrid, Spain) followed by density gradient centrifugation over Ficoll-Paque (Thermo Fisher Scientific, Waltham, MA, United States) at 540*g* for 20 min at room temperature (R/T). Adipose tissue lipoaspirates were first mechanically disaggregated using tweezers and a scalpel, enzymatically digested in DMEM medium containing 2 mg/mL collagenase type I (Roche Diagnostics, Basel, Switzerland) for 45 min at 37°C in a rotational stirrer, filtered through 40-μm nylon cell strainers (BD Biosciences, San Jose, CA, United States) and centrifuged at 540*g* for 10 min.

**Figure 1:**
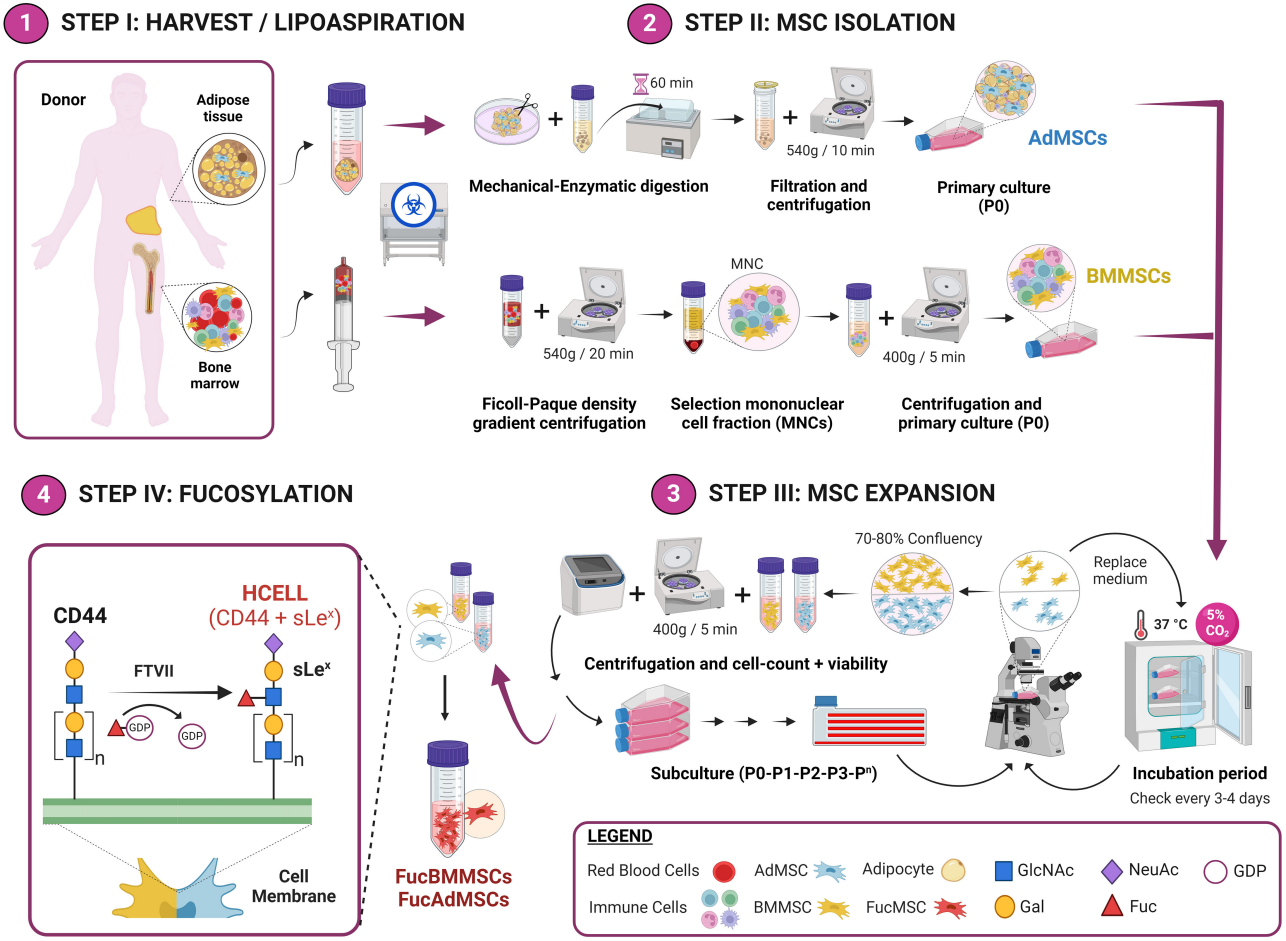
Experimental workflow of the methodology used for the isolation and exofucosylation of human MSCs for allogeneic clinical use. Bone marrow and adipose tissue samples were obtained from bone marrow harvest and lipoaspiration, respectively (Step I). After, BMMSCs and AdMSCs were isolated and expanded (Steps II and III), and subsequently exofucosylated to enforce expression of HCELL, a CD44 glycoform that is a potent E-selectin ligand (Step IV). GlcNAc, N-Acetylglucosamine; Gal, galactose; NeuAc, neuraminic acid; Fuc, fucose. Created by Biorender.

After centrifugation, mononuclear cells from bone marrow (BM) or adipose tissue (Ad) samples were collected, washed with Dulbecco’s phosphate-buffered saline (DPBS) (Sigma-Aldrich, St. Louis, MO, United States), plated in 175-cm^2^ culture flasks (NUNC, Thermo Fisher Scientific) at 1.6x10^5^ cells/cm^2^ in Alpha Minimum Essential Medium (MEM; Thermo Fisher Scientific) containing10% fetal bovine serum (FBS; Gibco, Waltham, MA, United States), 1% GlutaMAX (Thermo Fisher Scientific) and 1% penicillin/streptomycin (Lonza, Basel, Switzerland) and incubated at 37°C and 5% CO_2_ (**Figure 1**, Steps I and II).

For culture passage, cells were lifted by treatment with TrypLE Express dissociation reagent (Gibco) for 5 min at 37°C. Both BMMSCs and AdMSCs were grown to a maximum of 70-80% confluence at each culture passage and expanded up to passages 3-4 for the subsequent experiments (**Figure 1**, Step III).

### MSC exofucosylation

Human BMMSCs and AdMSCs were exofucosylated as previously reported (21). Briefly, cells were resuspended at 2x10^7^ cells/mL in the exofucosylation reaction buffer composed of Hanks´ Balanced Salt Solution (HBSS) (Thermo Fisher Scientific) containing 40 μg/mL fucosyltransferase VII (FTVII) (Bio-Techne, R&D Systems, Minneapolis, MN, United States), 10 mmol/L HEPES (Merck Millipore, Burlington, MA, United States), 0.1% human serum albumin (HSA; Albutein, Grifols, Barcelona, Spain) and 1 mmol/L guanosine 5’-diphosphate (GDP)-fucose (Sigma-Aldrich), and treated at 37°C for 1 hour with gentle shaking (**Figure 1**, Step IV). After, treated MSCs were collected by centrifugation and washed twice with DPBS. Cell viability after exofucosylation was assessed by trypan blue exclusion (normally more than 90% live cells), and exofucosylation efficacy was evaluated using the phycoerythrin-labeled anti-sLe^X^ monoclonal antibodyHECA-452 (BD Biosciences) by flow cytometry.

### Cryopreservation of exofucosylated human MSCs in different freezing solutions and cell densities

Five freezing solutions were evaluated for optimizing cryopreservation conditions of fucosylated human BMMSCs (FucBMMSCs) and AdMSCs (FucAdMSCs). These solutions were made as follows: 1) saline solution (0.9% NaCl; Braun, Frankfurt, Germany) containing 10% dimethyl sulfoxide (DMSO; CryoSure, WAK-Chemie Medical GmbH, Steinbach, Germany) and 2% HSA; 2) saline solution containing 5% DMSO, 5% polyethylene glycol (PEG; Sigma-Aldrich) and 2% HSA; 3) saline solution containing 7.5% propylene glycol (PG; Sigma-Aldrich), 2.5% PEG and 2% HSA; 4) NutriFreez^®^ D10 cryopreservation medium, a commercial serum-free cryopreservation solution containing methylcellulose, 10% DMSO and other undisclosed ingredients (Biological Industries, Cromwell, CT, United States); and 5) CryoStore^®^CS10, an animal component-free cryopreservation medium containing 10% DMSO and other undisclosed ingredients (STEMCELL Technologies, Vancouver, Canada).

**Figure 2:**
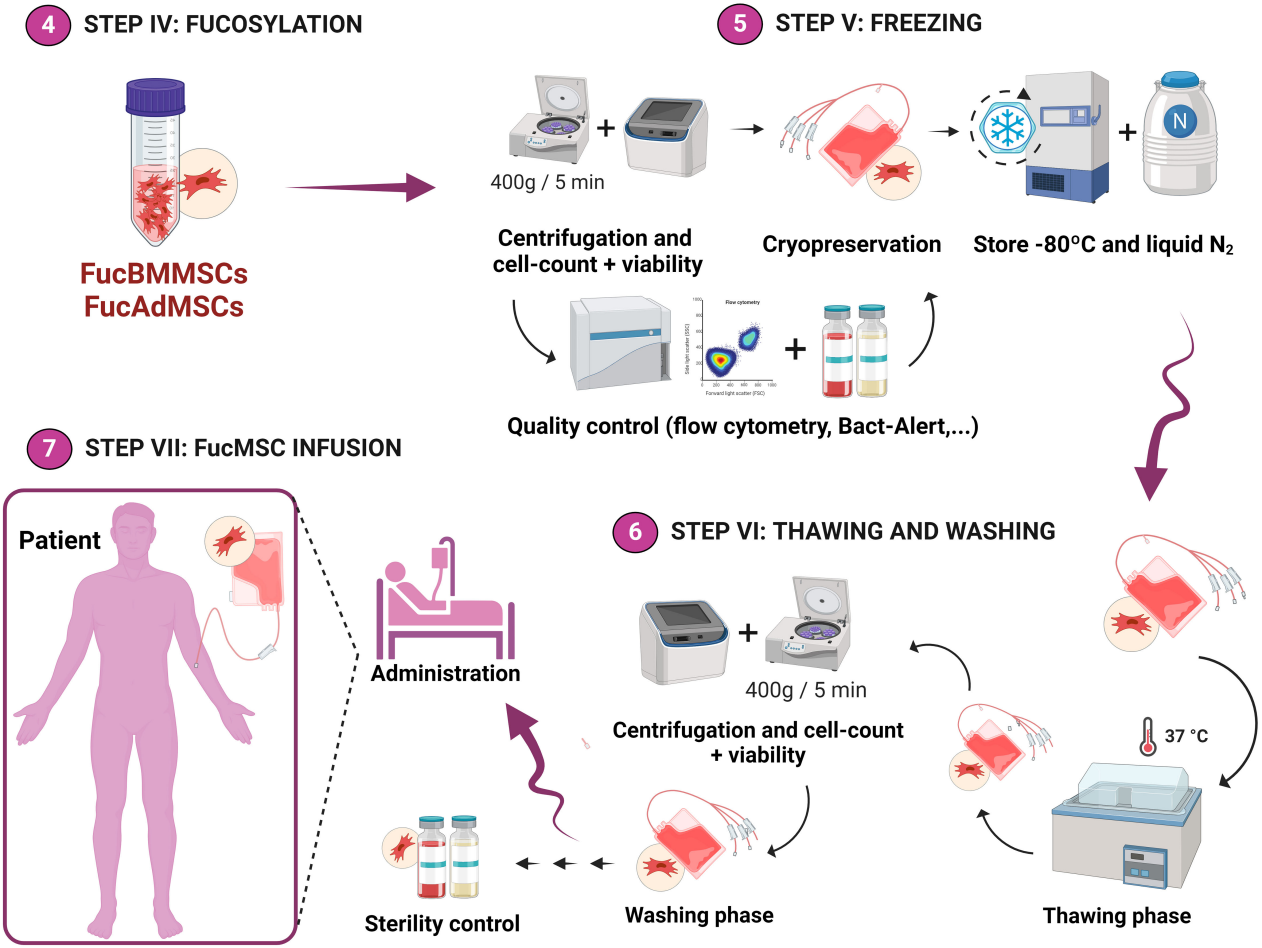
Experimental workflow of the methodology used for the cryopreservation of exofucosylated human MSCs for allogeneic clinical use. After analysis of cell viability and exofucosylation efficacy by flow cytometry, FucBMMSCs and FucAdMSCs were cryopreserved in different freezing solutions and cell densities (as detailed in Materials and Methods) and stored in liquid nitrogen (Step V). Finally, FucBMMSCs and FucAdMSCs cryovials (or cell freezing bags for large volumes) are typically thawed in a water bath at 37°C and washed (Step VI), being available for immediate allogeneic patient treatment (Step VII). Created by Biorender.

After exofucosylation, FucBMMSCs and FucAdMSCs were resuspended in ice-cold freezing solutions at 2x10^6^ cells/mL or 5x10^6^ cells/mL, transferred to 2 mL cryovials, stored at -80°C overnight in a freezing container (Corning CoolCell^®^; Corning Inc., Corning, NY, United States) to gradually decrease the temperature (1°C/min cooling rate) and then stored in liquid nitrogen for one month (**Figure 2**, Steps IV and V). Before being used in the different experiments, FucBMMSCs and FucAdMSCs cryovials were immediately placed in a water bath at 37°C and the cell suspensions were then rapidly washed with 10 volumes of a thawing solution composed of saline solution containing 2.5% HSA and 5% anticoagulant citrate-dextrose solution, solution A (ACD-A) (Grifols), centrifuged at 400*g* for 5 min and then gently resuspended in growth culture medium for complete cryopreservant removal (**Figure 2**, Step VI), being available for immediate allogeneic patient treatment (**Figure 2** **,** Step VII**)**.

### Analysis of cell viability and MSC immunophenotype

Cell viability of just thawed cryopreserved MSCs (without being cultured) was assessed by Annexin-V and 7-AAD staining (Immunostep, Salamanca, Spain) following the manufacturer´s instructions. Briefly, 1x10^5^ cells were resuspended in 100 μL 1X Annexin-V buffer followed by incubation with 5 μL FITC-labeled Annexin-V and 5 μL 7-AAD for 15 min in the dark at R/T. To evaluate MSC immunophenotype, just thawed FucBMMSCs and FucAdMSCs were stained with fluorescence-conjugated monoclonal antibodies for the cell markers CD73, CD90, CD105, CD14, CD20, CD34 and CD45 (Human MSC Phenotyping Kit; Miltenyi Biotec, Bergisch Gladbach, Germany) following the manufacturer´s instructions. Finally, cells were analyzed in an LSR Fortessa X-20 flow cytometer (Becton Dickinson, Franklin Lakes, NJ, United States) within 1h of staining. Non-specific fluorescence was measured using specific isotype monoclonal antibodies. All experimental conditions were evaluated in five separate experiments and analyzed using FlowJo software (FlowJo, LLC, Ashland, OR, United States).

### Analysis of cell proliferation kinetics

The cell proliferation kinetics of cryopreserved and thawed FucBMMSCs and FucAdMSCs was monitored by calculating the population doubling (PD) level up to 7 days in culture at 37°C using the formula: $\text{PD}=(\frac{1}{\log_{10}2})\times \log_{10}(\frac{Nt}{No})$ , where *No* is the number of viable cells at seeding and *Nt* the number of viable cells at harvest, as previously reported (20). Cumulative PD was calculated as the sum of individual PD obtained at the previous time points.

### Osteogenic and adipogenic differentiation assays

Cryopreserved and thawed FucBMMSCs and FucAdMSCs in the different experimental conditions were analyzed for their osteogenic and adipogenic differentiation potential using the StemMACS™ OsteoDiff and StemMACS™ AdipoDiff Media for human MSCs (Miltenyi Biotec), respectively, as per the manufacturer’s instructions. Briefly, MSCs were seeded in 24-well plates and cultured at 37°C until reaching 80-100% confluence. Afterward, the osteogenic or adipogenic differentiation media was added and changed every 2-3 days. After 21 days of culture, osteogenic differentiation was assessed by analyzing alkaline phosphatase (ALP) activity and the formation of calcified matrix culture deposits. For ALP activity, cells were fixed with 4% paraformaldehyde in PBS for 10 min at R/T and stained with SigmaFAST™ BCIP-NBT (Sigma-Aldrich) for 10 min at R/T, whereas calcified matrix culture deposits were detected after incubation with Alizarin Red staining solution (Sigma-Aldrich) for 30 min at R/T. For adipogenic differentiation, cells were cultured for 14 days and assessed by detection of intracellular lipid droplets with Oil Red O staining. Briefly, cells were fixed in 10% formalin for 10 min at R/T and stained with 0.3% Oil Red O (Sigma-Aldrich) in 2-propanol for 15 min at R/T. Finally, bright-field images of representative fields were acquired with a phase-contrast microscope (Nikon Eclipse Ti2, Nikon, Tokyo, Japan).

### Mitogen-stimulated T cell proliferation assays

Peripheral blood mononuclear cells from healthy donors were subjected to density gradient centrifugation over Ficoll-Paque (Thermo Fisher Scientific, Waltham, MA, United States) at 540*g* for 20 min at R/T. Then, T cells were isolated by magnetic separation using the Human Pan T Cell Isolation Kit (Miltenyi Biotec) following the manufacturer´s instructions. Isolated T cells were resuspended in RPMI 1640 medium (Gibco) containing 20% FBS, plated at 1x10^5^ cells/well in 96-well plates, stimulated to proliferate with 10 μg/mL phytohemagglutinin (PHA; Sigma-Aldrich) and co-cultured with decreasing MSC: T cell ratios 1:100, 1:50, 1:25 and 1:10. To prevent the MSC proliferation in the co-cultures but preserving the cell viability, MSCs were previously irradiated at 25 Gy. Finally, T cell proliferation in the co-cultures was measured 4 days later using an ELISA BrdU Colorimetric Kit (Roche Diagnostics), according to the manufacturer´s instructions.

For IFNγ preincubation, 20 ng/mL recombinant human IFNγ (Bio-Techne, R&D Systems) was added in the MSC culture medium for 24 hours and washed three times extensively with PBS before T cell addition.

### Senescence-associated-β-galactosidase assays

Cryopreserved and thawed FucBMMSCs and FucAdMSCs at passage 10, obtained after nine sequential freezing, thawing, and *in vitro* culture cycles, were seeded in 24-well plates at 1×10^5^ cells/well and allowed to attach O/N. Senescence was determined using the Senescence Cells Histochemical Staining Kit (Sigma-Aldrich) according to the manufacturer´s instructions. Briefly, 1X fixation buffer and 1X DPBS were made fresh from stocks and ultrapure distilled water on the day of the experiment. The staining mixture was made immediately before use by mixing 1 mL of the 10X staining solution, 125 μL of reagent B, 125 μL of reagent C, 250 μL of β-gal solution, 8.5 mL ultrapure distilled water (final volume: 10 mL), and then filtered through a 0.2-μm filter. The culture media were carefully aspirated off and cells were washed twice with DPBS. After, 1.5 mL of 1X fixation buffer was added to each well, incubated at R/T for 6 min, and washed twice with 1X DPBS. Finally, 1 mL of the prepared staining mixture was added to each well and each plate was sealed with parafilm. Dishes were then incubated at 37°C without CO_2_ O/N. The next day dishes were imaged under a bright-field inverted microscope (Nikon). The total number of SA-β-gal positive blue-stained FucMSCs and their relative number (percentage) respect to the total number of cells was measured in 20 non-overlapping and randomly chosen high power fields (HPF) per experimental condition.

### Statistical analysis

Statistical analysis was performed using GraphPad Prism v8 software (GraphPad, La Jolla, CA, United States). All data are presented as mean ± standard deviation (SD). One-way ANOVA with Tukey’s *post-hoc* multiple comparison tests was used to determine statistical differences between groups. A *P* value < 0.05 was considered statistically significant.

## Results

### Effect of the cryopreservation on the viability, the persistence of HCELL expression, and the immunophenotype of exofucosylated human MSCs after thawing

**Figure 3:**
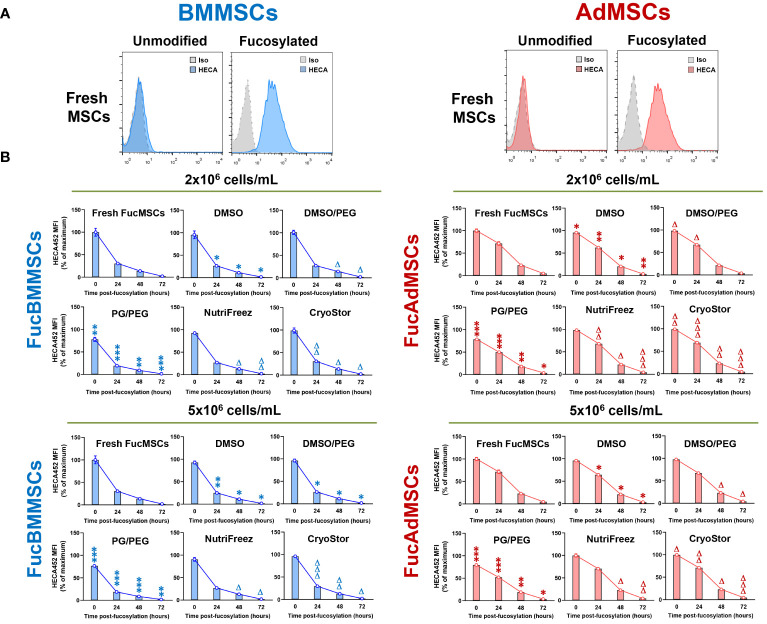
Kinetics of sLe^X^ surface expression after exofucosylation, cryopreservation, and thawing in the different freezing solutions and conditions. **(A)** Human BMMSCs and AdMSCs were exofucosylated and analyzed by flow cytometry for expression of sLe^X^ using HECA-452 mAb. Untreated BMMSCs and AdMSCs did not stain with HECA-452, whereas treatment with FTVII and GDP-fucose yielded HCELL staining. Control isotype antibody staining is also shown (grey histograms). **(B)** After cryopreservation in the different freezing solutions at different cell densities (2x10^6^ or 5x10^6^ cells/mL), freshly thawed FucBMMSCs and FucAdMSCs were seeded and cultured at 37°C for the indicated times, detached, and analyzed for sLe^X^ expression by flow cytometry as above. MFI, Mean fluorescence intensity. Data represent the mean ± SD from n=5 experiments and were analyzed using one-way ANOVA followed by Tukey’s *post-hoc* comparison test. sLe^X^ expression was significantly downregulated compared to Fresh FucMSCs that were not cryopreserved but cultured in the same conditions (i.e., Fresh FucMSCs), *p<0.05, **p<0.01 or ***p<0.001, respectively, or significantly upregulated compared to FucMSCs cryopreserved in the DMSO solution and thawed, Δ*p*<0.05, ΔΔ*p*<0.01 or ΔΔΔ*p*<0.001, respectively.

**Figure 4:**
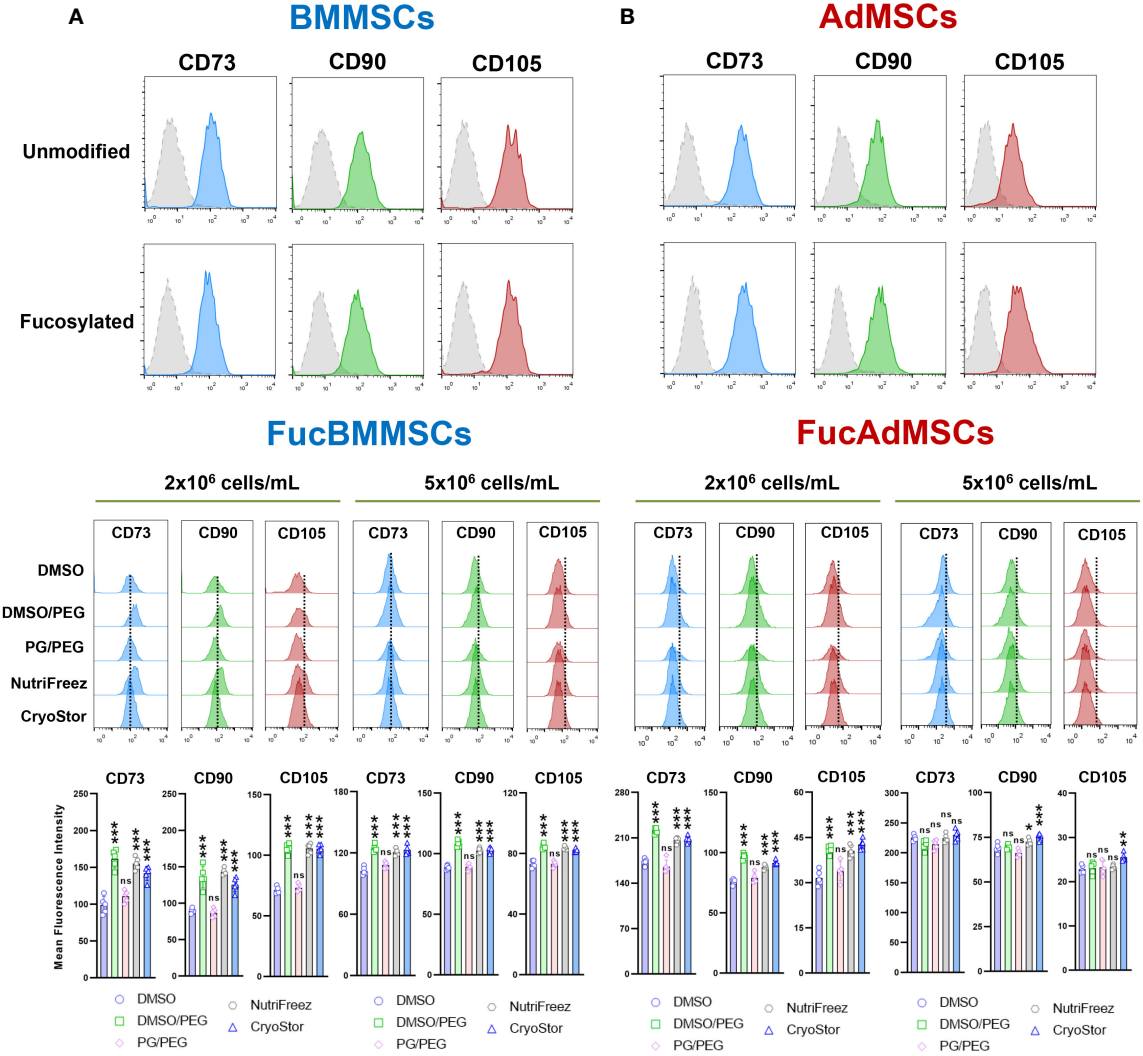
Immunophenotypic analysis of human exofucosylated BMMSCs and AdMSCs. **(A)** BMMSCs or **(B)** AdMSCs were modified by exofucosylation and immunophenotyped by flow cytometry. More than 95% of FucMSCs, and similar to unmodified MSCs, expressed the characteristic MSC surface markers CD73, CD90, and CD105 as defined by ISCT, and less than 2% showed expression of the hematopoietic markers CD14, CD20, CD34, and CD45 (not shown). Control isotype antibody staining (grey histograms) is also shown (upper histograms). Middle histograms represent the expression of each MSC marker displayed by cryopreserved and immediately thawed FucMSCs at the different freezing solutions and cell densities and are representative from n=5 experiments. Dotted lines represent the mean fluorescence intensity for each marker in Fresh FucMSCs but not cryopreserved. The mean fluorescence intensity of each MSC marker (mean ± SD from n=5 independent experiments) was significantly higher compared to the DMSO conditions, *p<0.05, **p<0.01, or ***p<0.001, respectively. ns, not significant. Data were statistically analyzed using one-way ANOVA followed by Tukey’s *post-hoc* comparison test.

After isolation and culture, human BMMSCs and AdMSCs were subjected to cell surface glycoengineering by glycosyltransferase-programmed stereosubstitution (*i.e.*, exofucosylation) using the α(1,3)-fucosyltransferase VII (FTVII) and its donor substrate GDP-fucose to modify CD44 to HCELL, a fucosylated glycoform of CD44 with strong binding affinity for E-selectin, as previously described (29, 30). As is shown in **Figure 3A**, flow cytometry analysis confirmed that both unmodified human BMMSCs (left) and AdMSCs (right) did not natively express the tetrasaccharide sLe^X^ as demonstrated by the absence of reactivity with the monoclonal antibody HECA-452. By contrast, after modification of MSCs by exofucosylation (i.e., FucBMMSCs and FucAdMSCs), more than 95% of the cells displayed a substantial upregulation of surface sLe^X^ expression, as evidenced by intense positive HECA-452 staining (**Figure 3A**), as previously reported for human and mouse BMMSCs and AdMSCs (20, 22, 29). Importantly, the exofucosylation protocol did not alter the surface expression of typical MSC markers, as freshly exofucosylated FucBMMSCs and FucAdMSCs, and similar to unmodified counterparts, were uniformly positive for CD73, CD90, and CD105 (**Figures 4A, B**, upper histograms) and negative for CD14, CD20, CD34, and CD45 (not shown).

To evaluate the impact of cryopreservation on the persistence of enforced sLe^X^ expression after exofucosylation, cryopreserved FucBMMSCs and FucAdMSCs in the different freezing solutions and conditions were thawed and re-cultured at 37°C up to 72h. Fresh FucBMMSCs and FucAdMSCs that were not cryopreserved (i.e., “Fresh FucMSCs”) displayed the maximum level of sLe^X^ expression immediately after exofucosylation, decreased to ~30% and ~71% after 24h of culture, respectively, and returned to basal absence of sLe^X^ displayed after 72h (**Figure 3B**). Importantly, we also observed that sLe^X^ levels were significantly retained in cryopreserved and just thawed FucBMMSCs and FucAdMSCs. However, some differences were found between the different freezing solutions in terms of the persistence of sLe^X^ expression detection over the culture time. FucBMMSCs and FucAdMSCs cryopreserved in the DMSO and PG/PEG solutions displayed significant decreases of sLe^X^ expression compared to levels shown by freshly fucosylated MSCs at different time points (*p<0.05, **p<0.01, ***p<0.001). However, FucMSCs cryopreserved in DMSO/PEG, and mainly in both NutriFreez and CryoStor, displayed sLe^X^ levels significantly higher than those observed with the DMSO solution (Δ*p*<0.05, ΔΔ*p*<0.01, ΔΔΔ*p*<0.001). Again, these differences were more pronounced when FucBMMSCs were cryopreserved at a cell density of 2x10^6^ cells/mL compared to 5x10^6^ cells/mL, and FucAdMSCs at 5x10^6^ cells/mL compared to 2x10^6^ cells/mL, respectively (**Figure 3B**).

Furthermore, we investigated whether cryopreservation and subsequent thawing could affect the expression of positive MSC surface markers on FucBMMSCs and FucAdMSCs (**Figures 4A, B**, middle histograms, and bottom graphs). In general, immediately thawed FucBMMSCs and FucAdMSCs showed a slight but significant downregulation of CD73, CD90, and CD105 compared to the levels shown by Fresh FucMSCs (represented by a dashed within the histograms). However, there were again some significant differences when comparing the different freezing solutions. Compared to cells that were cryopreserved in DMSO, FucMSCs cryopreserved in the DMSO/PEG, NutriFreez and CryoStor solutions showed significant higher maintenance of CD73, CD90, and CD105 expression levels (*p<0.05, ***p<0.001). This finding was observed in both FucBMMSCs and FucAdMSCs that had been cryopreserved at either 2x10^6^ cells/mL or 5x10^6^ cells/mL cell density (**Figures 4A, B**, middle histograms and bottom graphs).

**Figure 5:**
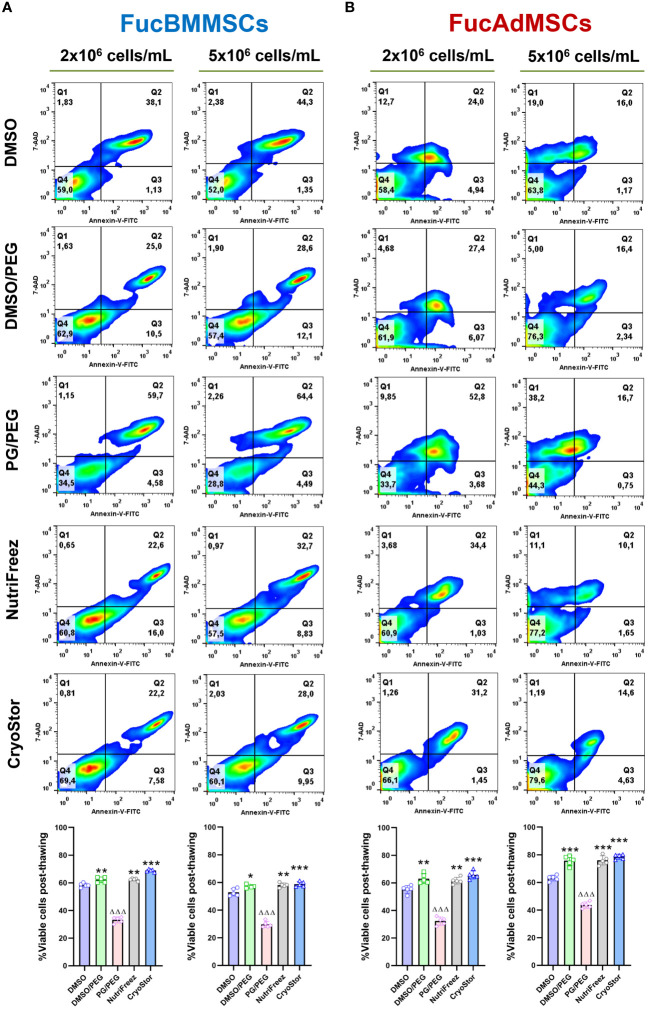
Effects of cryopreservation on the viability of human FucBMMSCs and FucAdMSCs using the different cryopreservation solutions and cell densities. Cell viability of cryopreserved and immediately thawed FucBMMSCs **(A)** or FucAdMSCs **(B)** was analyzed by Annexin-V and 7-AAD staining. Representative dot-plots from n=5 experiments are shown. Graphs represent the mean ± SD from n=5 independent experiments, and data were analyzed using one-way ANOVA followed by Tukey’s *post-hoc* comparison test. Percentages of viable cells (Annexin-V^-^ and 7-AAD^-^) were significantly higher compared to the DMSO conditions, *p<0.05, **p<0.01, or ***p<0.001, respectively, or significantly lower than the DMSO conditions, ΔΔΔ*p*<0.001.

The effects of different conditions of cryopreservation on the viability of immediately thawed FucMSCs were also investigated through flow cytometry by using Annexin-V and 7-AAD staining (**Figure 5**). As previously reported for native human MSCs (27, 31, 32), the viability of FucBMMSCs and FucAdMSCs cryopreserved in DMSO (*i.e*, the most widely used freezing solution for human MSCs) dropped to ~50-60% (**Figure 5**). However, the percentage of live cells previously cryopreserved in DMSO/PEG, NutriFreez, and CryoStor solutions were significantly higher (at 57.2%-68.9% for FucBMMSCs, and 61.6%-78.6% for FucAdMSCs, respectively) compared to those observed in DMSO (at 52.9%-58.3% for FucBMMSCs, and 55.2%-63.1% for FucAdMSCs, respectively) (*p<0.05, **p<0.01, ***p<0.001), while in PG/PEG were significantly lower (at 29.6%-33.2% for FucBMMSCs, and 32.3%-43.6% for FucAdMSCs, respectively) (ΔΔΔ*p*<0.001). In general, slightly higher viability percentages were obtained when FucBMMSCs were cryopreserved at a cell density of 2x10^6^ cells/mL compared to 5x10^6^ cells/mL, and FucAdMSCs at 5x10^6^ cells/mL compared to 2x10^6^ cells/mL, respectively (**Figure 5**).

Altogether, these data suggest that FucBMMSCs and FucAdMSCs cryopreserved in DMSO/PEG, NutriFreez, and CryoStor, at 2x10^6^ cells/mL and 5x10^6^ cells/mL, respectively, showed better cell viability, persistence of expression of sLe^X^ and MSC markers after thawing than DMSO and PG/PEG solutions.

### Morphology and cell proliferation kinetics of human FucBMMSCs and FucAdMSCs after cryopreservation

**Figure 6:**
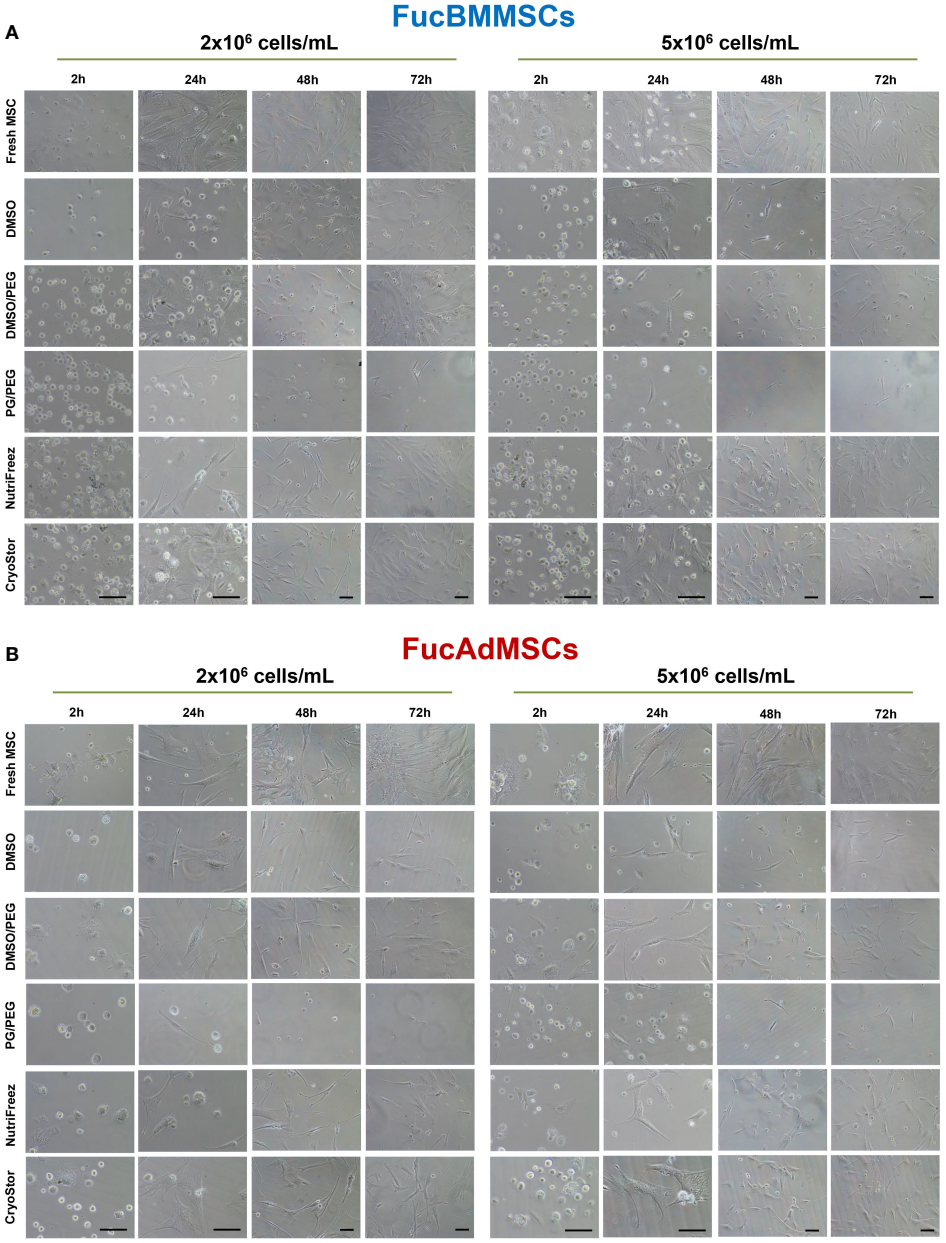
Analysis of cell adherence and morphology of cryopreserved and freshly thawed FucMSCs. Cryopreserved FucBMMSCs **(A)** or FucAdMSCs **(B)** in the different freezing solutions and conditions were thawed, washed, seeded at 3x10^3^ viable cells/cm^2^, and cultured up to 72h at 37°C. Representative images of cultures were obtained under an inverted phase-contrast microscope at the indicated times. Images at 2h and 24h: magnification x200. Images at 48h and 72h: magnification x100. Scale bar: 100 μm.

To analyze whether cryopreservation could affect FucMSC morphology directly after thawing, cryopreserved cells in the different solutions and conditions were seeded directly after thawing and cultured for up to 72h. As is shown in **Figures 6A, B**, Fresh FucBMMSCs and FucAdMSCs (i.e., not cryopreserved) displayed a rapid plastic adherence, cell spreading, and the typical fibroblast-like morphology of MSCs only after 24h of culture. However, as expected, cryopreserved and thawed FucMSCs displayed a delayed attachment compared to Fresh FucMSCs. Among all the freezing solutions tested, CryoStor, NutriFreez, and DMSO/PEG were those showing better and faster cell attachment and spreading, reaching 70-80% confluence after 72h of culture, mainly when FucBMMSCs and FucAdMSCs were cryopreserved at 2x10^6^ cells/mL and 5x10^6^ cells/mL, respectively. On the contrary, DMSO, and especially PG/PEG, showed a remarkable impairment of the reactivation of MSC growing in culture.

**Figure 7:**
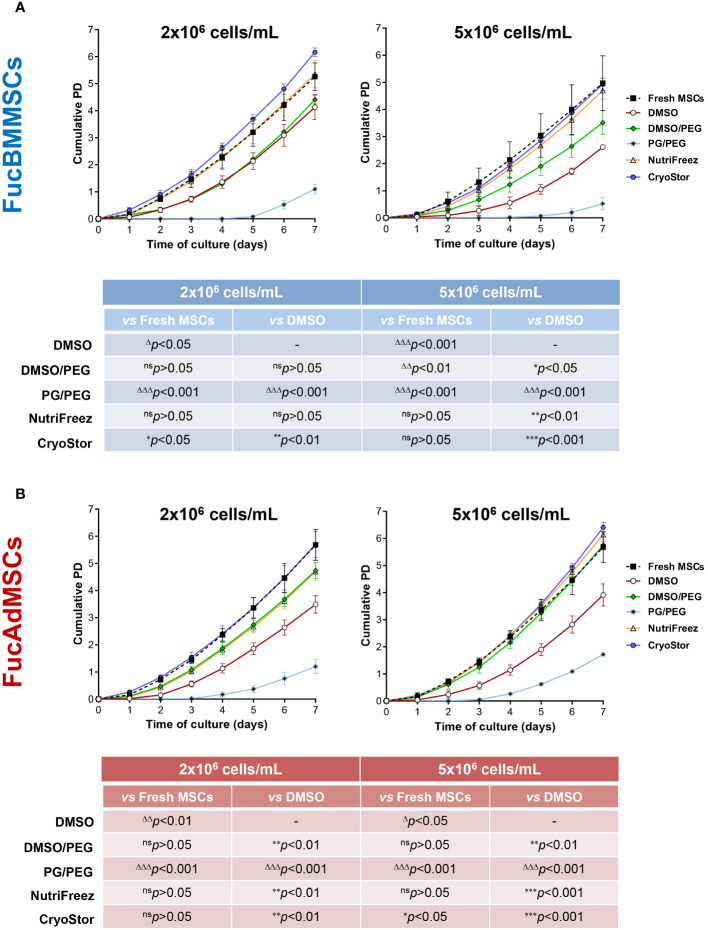
Analysis of cell proliferation kinetics of cryopreserved and freshly thawed FucMSCs. Cryopreserved FucBMMSCs **(A)** or FucAdMSCs **(B)** in the different freezing solutions and cell densities were thawed, washed, seeded at 3x10^3^ viable cells/cm^2^, and cultured for up to 7 days at 37°C. At the indicated time points, cells were detached and counted, and cumulative population doubling (PD) numbers were calculated using the formula specified in the Material and Methods section. Results are presented as mean ± SD from n=3 experiments and were analyzed using one-way ANOVA followed by Tukey’s *post-hoc* comparison test. Cumulative PD numbers were significantly higher (*p<0.05, **p<0.01, or ***p<0.001) or significantly lower (Δ*p*<0.05, ΔΔ*p*<0.01, or ΔΔΔ*p*<0.001) compared to Fresh FucMSCs that were not cryopreserved but cultured in same conditions (*i.e*., Fresh FucMSCs), or compared to FucMSCs cryopreserved in the DMSO solution, respectively. ns, not significant.

In addition, proliferation kinetics of the different cryopreserved and thawed FucMSCs were investigated using the cumulative population doubling (PD) calculation up to 7 days of culture. FucBMMSCs and FucAdMSCs cryopreserved in CryoStor solution at 2x10^6^ and 5x10^6^ cells/mL, respectively, exhibited significantly higher cumulative PD as compared to Fresh FucMSCs (i.e., not cryopreserved) (*p<0.05) and to the DMSO solution (**p<0.01, ***p<0.001), while NutriFreez displayed comparable cumulative PD levels to Fresh FucMSCs (**Figures 7A, B**).

### Impact of the exofucosylation and the cryopreservation on osteogenic and adipogenic differentiation capacities of human MSCs

**Figure 8:**
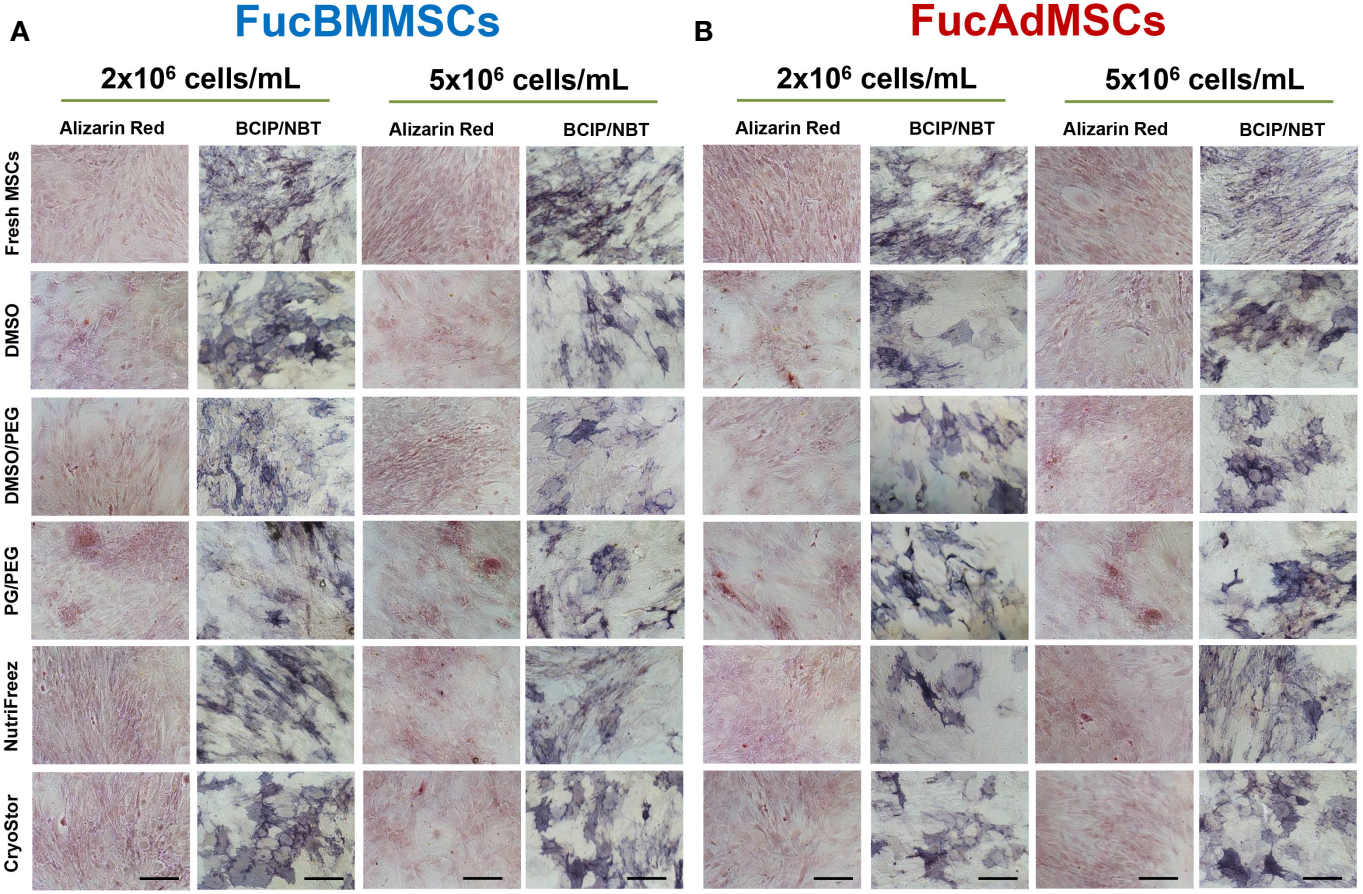
Impact of cryopreservation on the osteogenic differentiation capacity of human exofucosylated BMMSCs and AdMSCs. **(A)** BMMSCs or **(B)** AdMSCs were exofucosylated, cryopreserved in different conditions, thawed, and seeded to evaluate their osteogenic differentiation capacity. After 21 days of culture with osteogenic differentiation medium formation of calcified matrix deposits and alkaline phosphatase activity were evaluated by Alizarin Red and SigmaFAST™ BCIP-NBT staining, respectively. Representative results from n=3 differentiation experiments are shown. Magnification x200. Scale bar: 100 μm.

**Figure 9:**
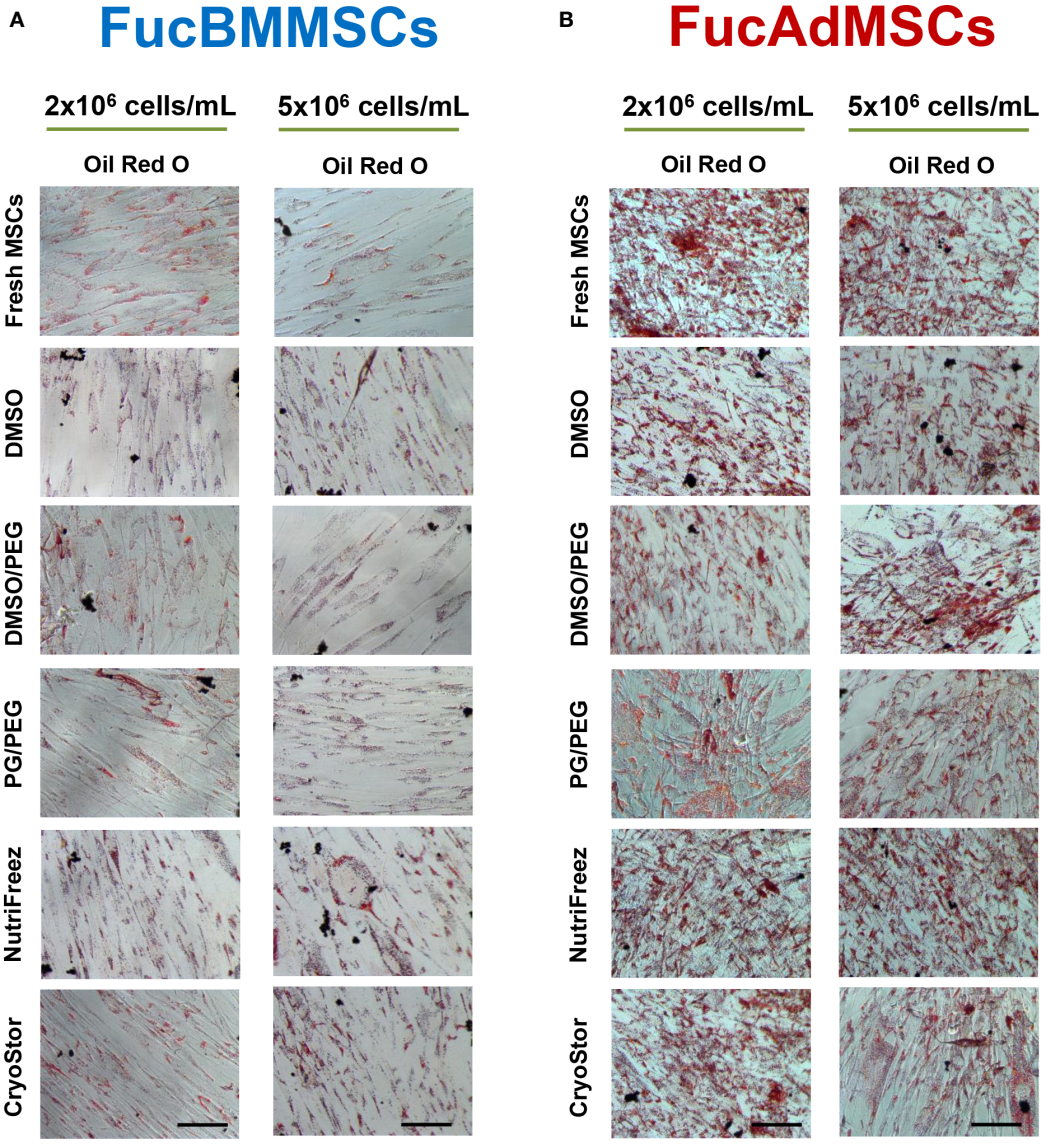
Impact of cryopreservation on the adipogenic differentiation capacity of human exofucosylated BMMSCs and AdMSCs. **(A)** BMMSCs or **(B)** AdMSCs were exofucosylated, cryopreserved in different conditions, thawed, and seeded to evaluate their adipogenic differentiation capacity. After 14 days of culture with adipogenic differentiation medium formation of intracellular lipid droplets were evaluated by Oil Red O staining. Representative results from n=3 differentiation experiments are shown. Magnification x200. Scale bar: 100 μm.

We studied whether exofucosylation and subsequent cryopreservation could affect the osteogenic (**Figure 8**) and adipogenic differentiation potential (**Figure 9**) of human FucBMMSCs and FucAdMSCs. For this purpose, cryopreserved and thawed FucMSCs were cultured for 21 days or 14 days in an osteogenic or adipogenic differentiation media, respectively, and subsequently subjected to osteogenic- or adipogenic-specific qualitative staining. Except for the time required to reach cell confluence when using some cryoprotectants and initiating the differentiation cultures, cryopreserved FucBMMSCs and FucAdMSCs in all conditions and thawed showed abundant mineral matrix depositions around the cells, positive alkaline phosphatase activity and lipid droplet formation comparable to those observed in Fresh FucMSCs. In addition, the cell density used for cryopreservation did not significantly alter the osteogenic and adipogenic differentiation capacities of human FucMSCs (**Figures 8A, B**, **9A, B**).

### Comparative evaluation of the immunomodulatory properties of cryopreserved and immediately thawed exofucosylated human MSCs

**Figure 10:**
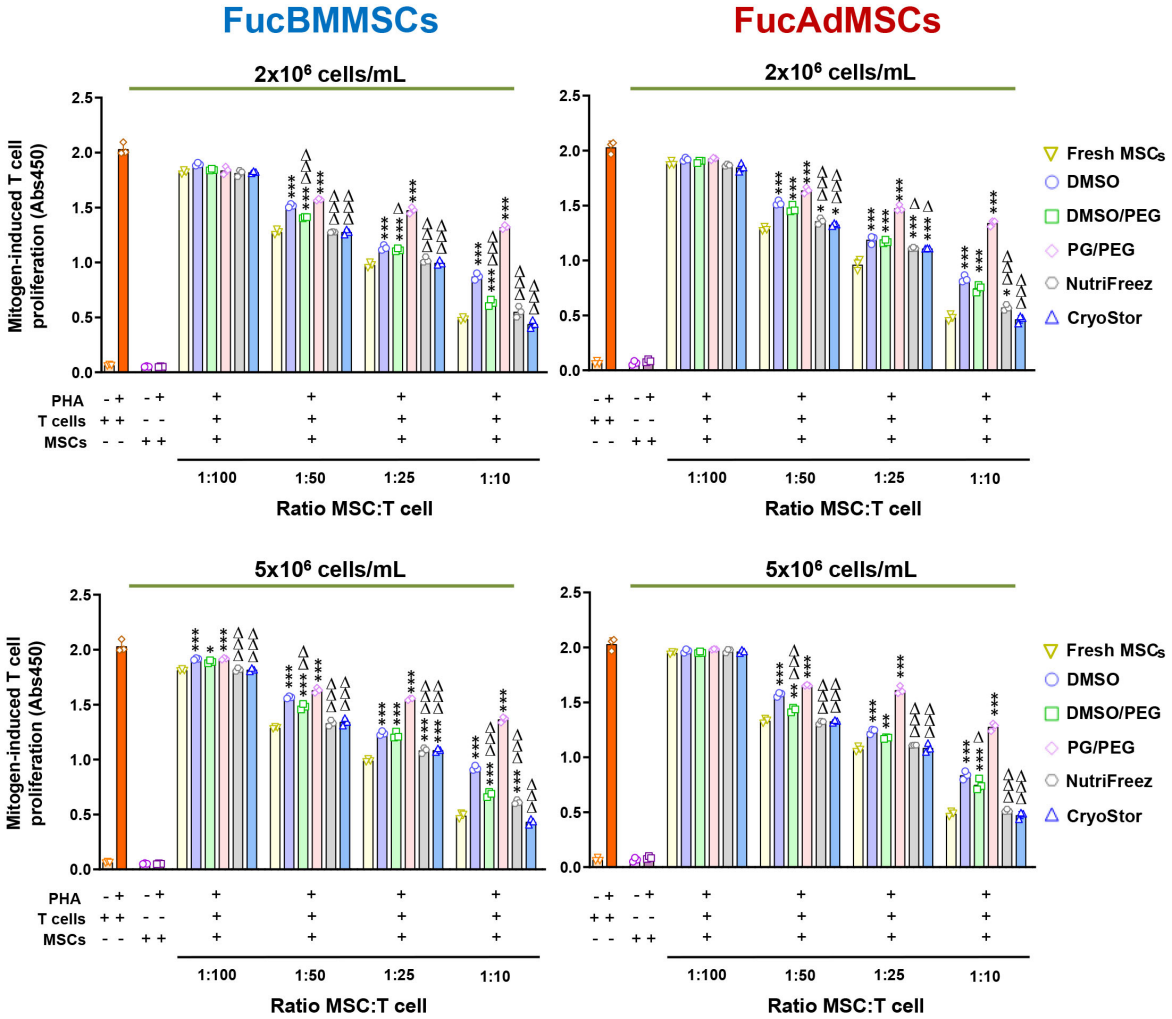
Immunomodulatory potential of exofucosylated human MSCs after cryopreservation and thawing. Phytohemagglutinin (PHA)-stimulated peripheral blood T cells were co-cultured with different ratios of thawed allogeneic FucBMMSCs or FucAdMSCs after being cryopreserved in the different freezing solutions and cell densities, or Fresh FucMSCs that were not cryopreserved (Fresh FucMSCs) as a positive control. Proliferation levels of T cells or MSCs cultured alone in the absence or presence of PHA are also shown. After 4 days of co-cultures, T cell proliferation was determined by BrdU incorporation (absorbance at 540 nm). Data represent the mean ± SD from n=3 experiments and were analyzed using one-way ANOVA followed by Tukey’s *post-hoc* comparison test. The PHA-induced proliferation of T cells was significantly higher compared to levels found with Fresh FucMSCs at each specific MSC: T cell ratio, *p< 0.05, **p<0.01, ***p<0,001, respectively, or significantly lower compared to levels found with FucMSCs cryopreserved in DMSO at each specific MSC: T cell ratio, Δ*p*<0.05, ΔΔ*p*<0.01 or ΔΔΔ*p*<0.001, respectively.

To assess whether cryopreservation could affect the immunomodulatory properties of FucMSCs, we analyzed the capacity of FucBMMSCs and FucAdMSCs to inhibit mitogen-stimulated T cell proliferation. Assays with human FucMSCs from different donors and cryopreserved at both 2x10^6^ and 5x10^6^ cells/mL showed that Fresh FucMSCs inhibited PHA-stimulated T cell proliferation in a dose-dependent manner (from a ratio of 1:100 to 1:10 MSC: T cell) more effectively than cryopreserved and thawed cells (**Figure 10**). However, FucBMMSCs and FucAdMSCs cryopreserved in NutriFreez or CryoStor at 2x10^6^ and 5x10^6^ cells/mL, respectively, showed an immunosuppressive effect comparable to that of Fresh FucMSCs at all MSC: T cell ratios. Regarding the other cryoprotective solutions, FucMSCs frozen in DMSO/PEG also showed a significantly higher immunosuppressive capacity than those cryopreserved in DMSO (**Figure 10**).

**Figure 11:**
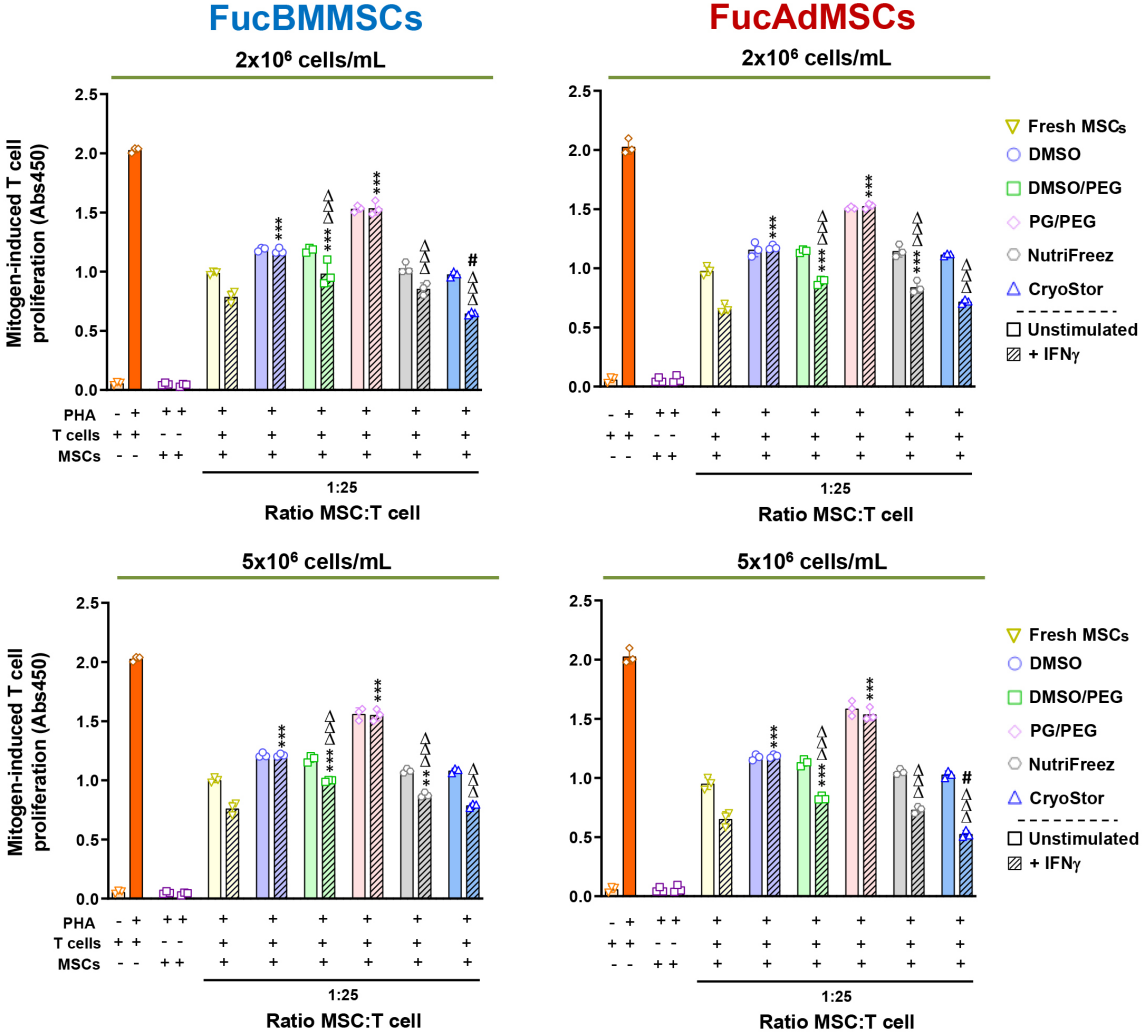
Immunomodulatory potential of exofucosylated human MSCs after cryopreservation, thawing, and IFNγ-prestimulation. Phytohemagglutinin (PHA)-stimulated peripheral blood T cells were co-cultured with different ratios of thawed unstimulated or IFNγ-pre-stimulated allogeneic human FucBMMSCs or FucAdMSCs after being cryopreserved in the different freezing solutions and cell densities, or fresh unstimulated or IFNγ-pre-stimulated FucMSCs that were not cryopreserved (Fresh FucMSCs) as a positive control. Proliferation levels of T cells or MSCs cultured alone in the absence or presence of PHA are also shown. After 4 days of co-cultures, T cell proliferation was determined by BrdU incorporation (absorbance at 540 nm). Data represent the mean ± SD from n=3 experiments and were analyzed using one-way ANOVA followed by Tukey’s *post-hoc* comparison test. The PHA-induced proliferation of T cells observed with cryopreserved, thawed and IFNγ-pre-stimulated FucMSCs was significantly higher compared to levels found with IFNγ-pre-stimulated Fresh FucMSCs at each specific MSC: T cell ratio, **p<0.01, ***p<0,001, or significantly lower compared to levels found with IFNγ-pre-stimulated Fresh FucMSCs at each specific MSC: T cell ratio, ^#^ *p*<0.05, or significantly lower compared to levels found with FucMSCs cryopreserved in DMSO at each specific MSC: T cell ratio, ΔΔΔ*p*<0.001, respectively.

Since DMSO-cryopreserved and thawed MSCs were previously reported to be refractory to IFNγ-preincubation to stimulate their immunosuppressive properties (31), we next studied whether this detrimental effect extended to non-DMSO cryopreservatives. As is shown in **Figure 11**, cryopreserved FucBMMSCs and FucAdMSCs in DMSO or PG/PEG solutions and subsequently stimulated with IFNγ were unable to significantly inhibit PHA-activated T cell proliferation compared to Fresh FucMSCs or cryopreserved FucMSCs in DMSO/PEG, NutriFreez and CryoStor counterparts. Strikingly, IFNγ-stimulated FucBMMSCs and FucAdMSCs cryopreserved in CryoStor at 2x10^6^ cell/mL and 5x10^6^ cells/mL, respectively, exhibited a significantly higher anti-mitogenic effect on T cell proliferation at a 1:10 MSC: T cell ratio compared to those observed with Fresh FucMSCs (^#^ *p*<0.05) (**Figure 11**).

Taken together, these results suggest that there are significant differences in the immunomodulatory capacity of cryopreserved FucMSCs in the context of T cell proliferation assays after mitogen stimulation, depending on the cryoprotectant used. Importantly, NutriFreez and CryoStor solutions increase the immunoregulatory activity beyond that observed with Fresh FucMSCs.

### Evaluation of senescence-associated β-galactosidase expression on cryopreserved exofucosylated human MSCs

**Figure 12:**
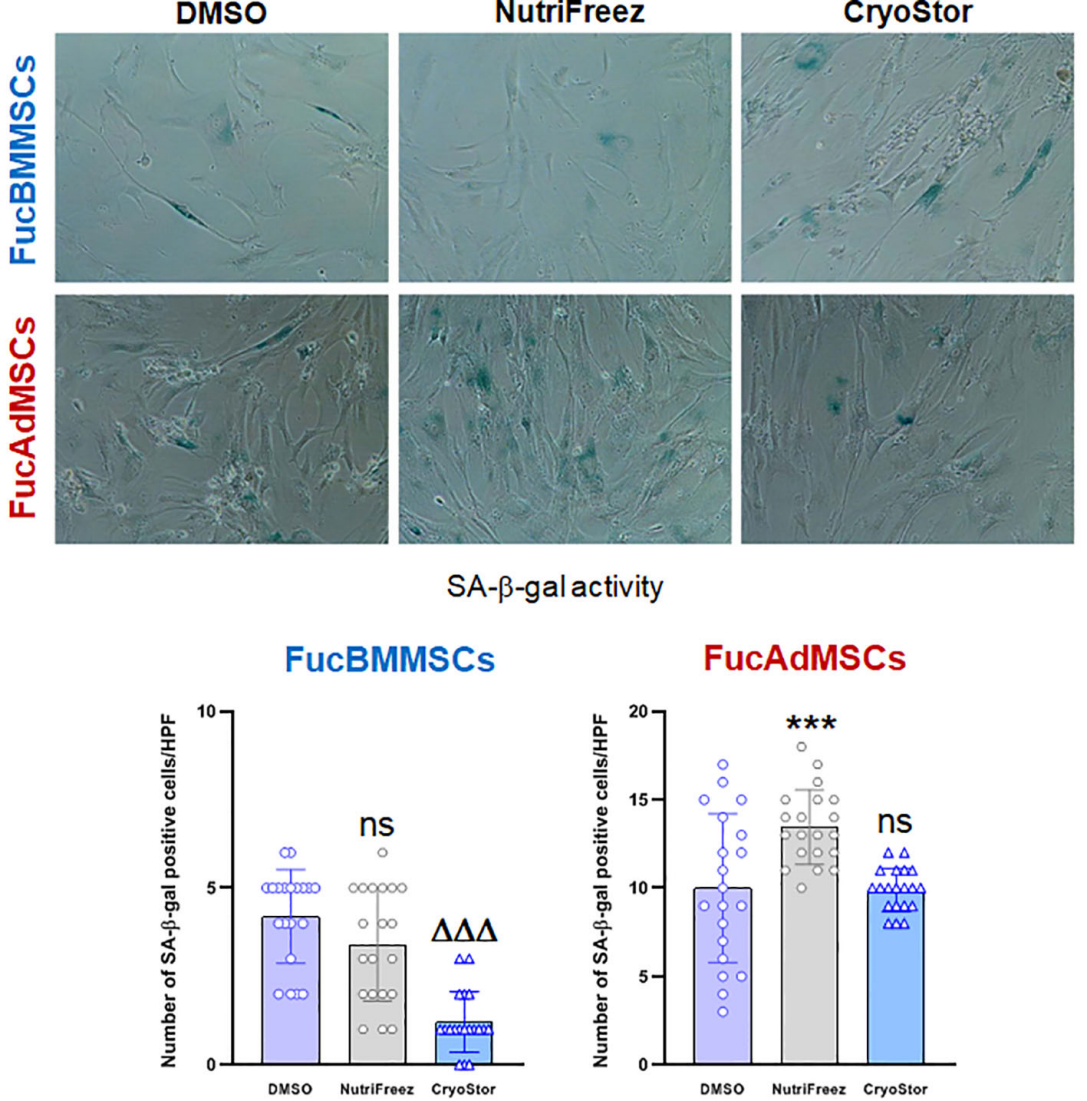
Expression of senescence-associated (SA)-β-galactosidase (gal) on cryopreserved and thawed human exofucosylated BMMSCs and AdMSCs. Representative images of SA-β-gal staining of FucBMMSCs and FucAdMSCs after successive cycles of cryopreservation in DMSO, NutriFreez, or CryoStor, thawing, and culture are shown. Magnification x200. The number of SA-β-gal positive blue stained cells was measured in 20 non-overlapping and randomly chosen high power fields (HPF) per experimental condition. Graphs represent the mean ± SD of the number of senescent cells per HPF. Data were statistically analyzed using one-way ANOVA followed by Tukey’s *post-hoc* comparison test. SA-β-gal positive cells number were significantly increased compared to the DMSO conditions, ***p<0.001, or significantly decreased compared to the DMSO conditions, ΔΔΔ*p*<0.001, respectively. ns, not significant.

To further investigate the relevance of cryopreservation on FucMSC senescence after *in vitro* culture, we analyzed senescence-associated (SA)-β-galactosidase (gal) expression in FucBMMSCs and FucAdMSCs at passage 10 after nine sequential freezing steps in DMSO, NutriFreez and CryoStor solutions, thawing and *in vitro* culture (**Figure 12**). Although low numbers of SA-β-gal positive cells were detected in all cultures, the levels of senescent cryopreserved FucBMMSCs in CryoStor and FucAdMSCs in NutriFreez were significantly decreased and increased, respectively, compared to the DMSO conditions (ΔΔΔ*p*<0.001, ***p<0.001) (**Figure 12**). Likewise, the percentage of SA-β-gal positive cells in relation to the total number of FucMSCs also showed the same trend (FucBMMSCs: DMSO (14.00% ± 2.3%), NutriFreez (10.3% ± 6.1%), and CryoStor (6.6% ± 3.37%); FucAdMSCs: DMSO (17.6% ± 11.8%), NutriFreez (25.9% ± 10.0%), and CryoStor (17.7% ± 7.5%) (data not shown).

## Discussion

Cryopreservation is the most effective method of preserving and transporting biological samples for clinical use and has been successfully used for the long-term storage and transport of a wide variety of cell types, and is considered the most effective method of cell preservation for further cell use (33, 34). This method depends largely on thermodynamic kinetics and osmotic balance, and also some important physical factors could influence cryopreservation success after cell recovery including the cooling rate, the composition of the freezing media, cryoprotectants nature, cell exposure time, the storage temperature, and the critical thawing step that include a washing step for cryopreservant removal (35–38). However, during cryopreservation cells frequently experience cryogenic damage due to temperature fluctuations during the freezing step, formation of intracellular ice crystals, osmotic injury, and actin cytoskeletal disruption (39, 40). To mitigate these adverse consequences, some cryoprotective agents are added to the freezing medium to maintain cell architecture, viability and properties. However, the exact nature and the level of cryoprotection shown by these compounds to a specific cell type are still poorly understood. For MSCs, the most widely used cryoprotective agent is dimethyl sulfoxide (DMSO), but other cryoprotectants (e.g., PEG, PG, polyvinylpyrrolidone, or methylcellulose) have also been employed, as well as different combinations of them or with other additives such as serum, human platelet lysate or albumin to improve both the viability and the biological properties of the post-thawed cells (41–44).

On the other hand, some modifications of the MSCs are necessary for improving their cellular efficiency as cellular therapies and they could alter the MSC conditions for cryopreservation and thawing. To date, numerous clinical trials have been carried out to investigate the use of MSC-based medicine products for the treatment of multiple pathologies showing very encouraging results. However, one of the main limitations of MSC therapy for systemic diseases, where intralesional, local administration of cells is not feasible, is achieving the engraftment of a sufficient number of MSCs in the affected tissue. Thus, different bioengineering strategies have emerged to improve the tropism of MSCs to those tissues where they need to exert their beneficial effects including genetic modification, cell surface engineering, ultrasound techniques, ligand conjugation, magnetic guidance, or *in vitro* priming (23, 45–48). Among these, MSC fucosylation is a method to enzymatically transform CD44 into HCELL, allowing MSCs to tether and migrate more efficiently to E-selectin-bearing endothelial beds, mainly in the skin, bone marrow, and inflamed tissues, and also to potentiate their anti-inflammatory properties (20, 22, 29). Importantly, this bioengineering strategy has already been used in phase I clinical trial in patients with osteoporosis, with preliminary results suggesting that the treatment is safe, feasible, and with some potential clinical benefit (ClinicalTrials.gov Identifier: NCT02566655) (49).

Importantly, some authors have reported that systemic administration of MSCs can induce some pro-thrombotic events due to MSC expression of tissue factor (TF/CD142), activation of the complement cascade and/or a non-specific innate immune response, an event termed immediate blood-mediated inflammatory response (IBMIR), which greatly affects the function and survival of MSCs when administered without anticoagulants (12, 25, 27, 50). While some recent studies claim that tissue factor is not a convincing marker to predict pro-thrombotic risk induced by MSCs (51), other authors indicate that cryopreserved MSCs, thawed and washed in the presence of human blood AB plasma stimulate a more intense IBMIR (27, 52). However, to date it has not been reported whether modification of MSCs by fucosylation and/or cryopreservation with different cryoprotective agents and conditions could affect IBMIR, or whether fucosylation could up- or downregulate TF expression in MSCs.

On the other hand, the manufacture of fucosylated MSCs is complex, expensive, and time-consuming, potentially limiting their clinical applications and the access to this bioengineered cell product for patients (21). One possible solution is the creation of large-scale cryobanks of allogeneic fucosylated MSCs from healthy donors at clinical doses that would have several clinical advantages including: 1) immediate “off-the-shelf” access to the advanced cell therapy product; 2) achievement of all quality testing before batch release and administration to the patient; 3) availability of extra clinical cell doses from the same donor to administer repeated doses to the patient if necessary; and 4) the possibility of transporting cryopreserved clinical doses to be administered to patients in other geographical areas, and facilitate the launching of multicenter clinical trials with a homogeneous cell therapy product.

In the present study we tested the most widely used cryoprotective agent and concentration, i.e. 10% DMSO, and found two other cryoprotectant solutions that significantly improve the properties of fucosylated MSCs, including their anti-inflammatory/immunomodulatory activity: NutriFreez and CryoStor. Post-thaw viability, cell morphology and plastic adherence, proliferation kinetics, immunophenotype, senescence, and persistence of surface sLe^X^ expression were significantly preserved when these commercially available freezing solutions were used when compared to those properties observed with DMSO. To our information, other previous studies have also found beneficial effects of CryoStor and methylcellulose-containing freezing media on cell viability and functions after cryorecovery in comparison with DMSO (53–55). Other authors have also investigated the effect of reducing the concentration of DMSO in the freezing media by mixing it with other cryoprotectants in different proportions such as hydroxyethyl starch, PEG, PG, or trehalose, and reported higher cell viability, proliferative capacity and trilineage differentiation potential after thawing (41, 56, 57). Our results also indicated that a combination of DMSO and PEG at 1:1 proportion (i.e., 5% DMSO and 5% PEG) improves some phenotypic characteristics and functions of FucMSCs after thawing, while 10% DMSO, and especially the combination of PG/PEG, showed a remarkable impairment of the reactivation of the anti-inflammatory/immunomodulatory properties of FucMSCs after thawing.

The cell density at freezing has also been reported as a critical factor that could influence cell behavior after thawing due to variations in viscosity, cell metabolism, and other physical properties of the freezing media. Some studies have evaluated different cell densities ranging from 1x10^5^ to 1x10^7^ cells/mL (58–60). In this respect, we observed some significant differences and better cell outcomes when FucMSCs from bone marrow were cryopreserved at 2x10^6^cells/mL and from adipose tissue at 5x10^6^ cells/mL, respectively. This finding suggests that cell density is a critical parameter to consider and should be validated prior to cryopreservation of each specific cell type to ensure optimal functional recovery of the cells.

Trilineage differentiation ability of MSCs (i.e., adipogenic, osteogenic, and chondrogenic) is another criterion of the International Society for Cellular Therapy (ISCT) guide for defining MSCs (61). In agreement with our results using FucMSCs, previous work evaluated the effects of cryopreservation on the differentiation potential of MSCs after thawing, concluding that, although some results are controversial, cryopreservation does not alter MSC osteogenic, adipogenic or chondrogenic differentiation potential (data not shown) (56, 62–65). In addition, previous studies from our group showed that fucosylated murine and human MSCs are able to efficiently differentiate into adipocyte, osteoblast and chondroblast cell lineages (20, 21).

Therapeutic applications of MSCs are largely based on their immunomodulatory properties. In most studies the cells are re-cultured for a short period of time after thawing to achieve reactivation since various studies have shown that these MSC properties are impaired directly after thawing (31, 66). Moreover, it is an important issue in the case of FucMSCs because the enzymatic modification is transient, so the cell reactivation step after several days in culture would cause the cells to recover their basal state (i.e., expressing their native CD44 glycoform levels), and as a consequence, a loss of their improved trafficking *in vivo*. In addition, it has been shown that cryopreserved MSCs have a lower ability to suppress T cell proliferation immediately post-thaw, which is fully restored after brief reactivation culture (27, 67). In this regard, our results indicate that both FucBMMSCs and FucAdMSCs cryopreserved in NutriFreez or CryoStor at 2x10^6^ or 5x10^6^ cells/mL, respectively, were able to suppress mitogen-stimulated T cell proliferation to the same extent as fresh, not cryopreserved FucMSC counterparts. Furthermore, IFNγ-stimulated FucMSCs that have been cryopreserved in these two solutions and conditions, display a significant higher immunomodulatory potency compared to fresh counterparts, in contrast to previous studies showing that cryopreserved and thawed MSCs display a loss of IFNγ responsiveness and reduced suppressive licensing as a result of heat-shock response (31). Thus, the previously observed lack of efficacy of cryopreserved and thawed MSCs before the infusion to the patients might be reverted using our optimized cryopreservation protocols and without the need to reactivate the cells after thawing. However, these results should be interpreted with caution since we only evaluated the immunosuppressive ability of FucMSCs to inhibit T cell proliferation. Indeed, it has been described that MSCs block B cell differentiation and secretion of different immunoglobulin isotypes (i.e., IgG1, IgG2 and IgM) while promoting the maturation of circulating myeloid dendritic cells (68, 69). Likewise, that systemically administered MSCs undergo apoptosis and are subsequently phagocytosed by both circulating and tissue-resident phagocytic cells, a mechanism termed efferocytosis, which triggers host-induced immunomodulation *in vivo* (70, 71). However, the effects of fucosylation and/or cryopreservation of MSCs on the regulation of these other immune effector mechanisms have not been reported and warrant further investigation.

In summary, our findings suggest that FucBMMSCs and FucAdMSCs cryopreserved in DMSO/PEG, and mainly in NutriFreez and CryoStor solutions, at 2x10^6^ cells/mL and 5x10^6^ cells/mL, respectively, could represent alternative cell therapy products to be used in patients directly after thawing or after a short reactivation time in culture. These conditions preserve sLe^X^ expression, maintaining their tropism to inflamed/injured tissue(s). Altogether, these results provide new perspectives on the design of optimized protocols for bioengineered MSCs. Although additional studies are needed, mainly to evaluate the *in vivo* therapeutic efficacy of cryopreserved and thawed FucMSCs, our research might serve as a reference for similar studies with other bioengineered cell types also employed in advanced therapies, such as hematopoietic stem cells, regulatory T cells, neural stem cells or iPSCs, that require a previous cryopreservation step during the manufacturing, storage, and/or transportation to optimize the clinical use and access of cellular therapeutics.

## Data availability statement

The raw data supporting the conclusions of this article will be made available by the authors, without undue reservation.

## Ethics statement

This study was carried out in accordance with the recommendations of the Guideline for Good Clinical Practice from the Institutional Review Boards of the Virgen de la Arrixaca University Hospital (Murcia, Spain). All donors gave written informed consent following the Declaration of Helsinki. The studies were conducted in accordance with the local legislation and institutional requirements. The human samples used in this study were acquired from primarily isolated as part of your previous study for which ethical approval was obtained. Written informed consent for participation was not required from the participants or the participants’ legal guardians/next of kin in accordance with the national legislation and institutional requirements.

## Author contributions

JG: Conceptualization, Data curation, Formal Analysis, Investigation, Methodology, Writing – review & editing. CB: Investigation, Methodology, Writing – review & editing. CM: Formal Analysis, Investigation, Writing – review & editing. AF: Formal Analysis, Investigation, Writing – review & editing. AG: Conceptualization, Methodology, Validation, Writing – review & editing. MB: Conceptualization, Methodology, Supervision, Writing – review & editing. MM: Conceptualization, Methodology, Writing – review & editing. AZ: Conceptualization, Data curation, Formal Analysis, Methodology, Supervision, Validation, Writing – original draft, Writing – review & editing. RS: Conceptualization, Data curation, Formal Analysis, Methodology, Supervision, Writing – review & editing. JM: Conceptualization, Data curation, Formal Analysis, Funding acquisition, Methodology, Resources, Supervision, Writing – original draft, Writing – review & editing. DG: Conceptualization, Data curation, Formal Analysis, Funding acquisition, Investigation, Methodology, Project administration, Resources, Software, Supervision, Validation, Visualization, Writing – original draft, Writing – review & editing.
